# Comparative Stability of Synthetic and Natural Polymeric Micelles in Physiological Environments: Implications for Drug Delivery

**DOI:** 10.3390/pharmaceutics17111439

**Published:** 2025-11-07

**Authors:** Hurriyet Polat, Merve Cevik Eren, Mehmet Polat, Kyle M. Koss, Onur K. Polat

**Affiliations:** 1 Department of Chemistry, Izmir Institute of Technology, Urla 35433, Izmir, Türkiye; hurriyetpolat@iyte.edu.tr (H.P.); mervecevik@iyte.edu.tr (M.C.E.); 2 Department of Chemical Engineering, Izmir Institute of Technology, Urla 35433, Izmir, Türkiye; mehmetpolat@iyte.edu.tr; 3 Department of Internal Medicine-Nephrology, The University of Texas Medical Branch, Galveston, TX 77555, USA; kmkoss@utmb.edu; 4 Department of Neurobiology, The University of Texas Medical Branch, Galveston, TX 77555, USA; 5 Sealy Institute for Drug Discovery, The University of Texas Medical Branch, Galveston, TX 77555, USA

**Keywords:** polymeric micelles, biopolymers, bio-polymeric micelles, drug delivery, micelle-stability, self–assembly

## Abstract

Polymeric micelles are widely studied as nanocarriers for hydrophobic drugs, yet their structural stability under physiological conditions remains a major limitation. This review provides a comparative evaluation of synthetic and natural polymeric micelles with a focus on their stability under dilution and in protein-rich environments. The discussion integrates thermodynamic and kinetic factors governing micelle integrity and examines how molecular composition, hydrophobic segment length, and core–shell modifications influence disintegration behavior. While synthetic micelles commonly collapse below their critical micelle concentration during intravenous administration, natural polymeric micelles, such as those derived from chitosan, alginate, or heparin, exhibit improved resistance to dilution but remain vulnerable to protein-induced destabilization. Strategies such as core or shell cross-linking, surface functionalization, and natural polymer coatings are reviewed as promising approaches to enhance circulation stability and controlled drug release. The work provides a framework for designing micellar systems with balanced biocompatibility, biodegradability, and robustness suitable for clinical drug-delivery applications.

## 1. Introduction

Therapeutic compounds with low water solubility often require carriers to ensure stable transport in aqueous fluids. Among the various delivery systems explored in the literature, polymeric beads, liposomal, and micellar systems have been extensively studied [1].

Surfactant-based micelles, in particular, are widely used due to their versatility and commercial availability [2,3,4,5,6,7,8,9,10,11,12,13,14,15,16]. In copolymeric micelles, the hydrophobic core serves as a nanoscale reservoir for lipophilic drug molecules, while the hydrophilic shell (corona) ensures water solubility [17,18,19,20,21,22,23,24]. Their relatively large size, tunable chemical structure, biocompatibility, and ability to mimic natural carriers such as viruses make copolymeric micelles excellent drug-delivery vehicles for various pharmaceutical applications [25].

Applications include medical diagnostic imaging, drug delivery and targeting, immunology, and gene therapy. For instance, polymeric micelles enhance medical imaging by enabling the accumulation of contrast agents at targeted sites, thereby improving signal intensity [9]. When used as blood-pool agents (BPAs) in computed tomography (CT), their extended circulation times allow for high-resolution arterial and venous mapping [26,27,28].

The use of copolymers in drug delivery is well-established. In a comprehensive review on micelle stability, Owen et al. [29] trace the use of polymeric micelles back to early work by Ringsdorf et al. [30,31]. The wide range of commercially available polymers provides extensive opportunities to design copolymers with tailored properties.

Hydrophilic blocks can range from polyethylene glycol (PEG) to polypeptide mimics such as poly-L-lysine (PLL) and poly(lactic-co-glycolic acid) (PLGA). Lipophilic blocks include poly(lactide)/polylactic acid (PLA), poly(ε-caprolactone) (PCL), and functional polymers like poly(2-methacryloyloxyethyl phosphorylcholine) (PMPC) and poly(2-allyloxymethyl-2-ethyltrimethylene carbonate) (PAOMEC) [31]. This diversity of building blocks allows for the design of micelles with a wide variety of structural and functional properties.

## 2. Formation of Micellar Drug-Delivery Vehicles

The tendency of polar water molecules to exclude nonpolar molecules leads to attraction and spontaneous association (self-assembly) of the hydrophobic sections of a copolymer in an aqueous environment. A balance between this attraction and the affinity of the polar hydrophilic sections of a copolymer towards water leads to the creation of self-assembled micellar structures of well-defined size and morphology. The position of the equilibrium on the concentration scale is termed the critical micelle concentration (CMC). The CMC is basically the minimum concentration required for the molecules to self-assemble and is typically around the micromolar concentration range.

**Figure 1:**
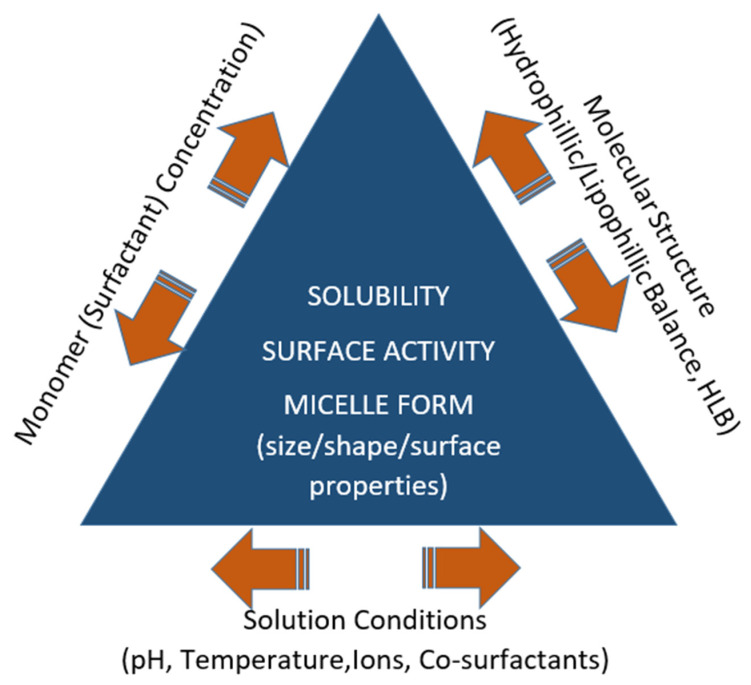
Factors that determine micelle formation and properties.

This equilibrium means that micellization is a reversible process. At concentrations lower than the CMC, the molecules of the surfactant are in the form of monomers and tend to accumulate at the air–water (or oil–water) interface. As the surfactant concentration is increased, both the bulk and the interface are saturated at a specific concentration, and the increased activity leads to self-aggregation (or self-assembly). However, for the polymeric micelles, the transition is not sharp and is highlighted by the gradual formation of dimers, trimers, etc. [32]. Hence, around the CMC, the micelles are loose and may contain some water in their core. They become more compact, durable, and smaller in size when the surfactant concentration is increased. Further increase in surfactant concentration may lead to changes in micellar morphology such as a transition from a spherical to cylindrical configuration. Micelle formation characteristics, and hence the CMC of a surfactant, are strongly influenced by the molecular structure, temperature, pH, presence of electrolytes, or other co-surfactants (Figure 1) [29,33,34,35].

The morphology of a micelle can differ depending on the chemical structure, chain length, and concentration of the copolymer, which permits tuning the size and shape of the micelle for a specific application. For example, rod-shaped micelles have better mechanical stability; round-shaped micelles provide good colloidal stability; lamella-shaped micelles allow better control of shape and size distribution; worm-shaped micelles have a high tendency to bind and coalesce [35,36,37,38]; and cylindrically shaped micelles of simple surfactants are widely employed to prepare soft templates in manufacturing micro-porous structures [39].

## 3. Requirements for Micellar Drug-Delivery Vehicles

For a micelle to function as a successful drug carrier, it must fulfill several requirements functions. The micelle must be biocompatible and benign to the biological environment. In other words, it must properly perform its intended functions in the tissue through induced reactions without inducing unacceptable toxic, immunogenic, thrombogenic, or carcinogenic responses. To achieve this, the carrier system should possess physicochemical properties that render it non-immunogenic and compatible with the host immune system. It must show the right amounts of immunostimulatory (e.g., to prevent secondary tumor formation by stimulating the immune system) and immunosuppressive (e.g., to allow the acceptance of foreign tissues in organ transplant patients) properties. The main factor in determining the immuno-compatibility of a carrier is basically related to its surface properties [40], which form the basis of devising various methods such as polyethylene glycol addition or PEGylation [41] to modify the carrier system to achieve compatibility. The surface properties of the carrier also determine its interaction, and hence compatibility, with native blood components which may influence its efficiency and may even render it inoperative.

The biological response and overall suitability of a given material are context-dependent and may differ across applications and tissue environments. For example, biodegradable poly(lactic-co-glycolic acid) (PLGA) polymeric-based nano-and microspheres, which offer a well-characterized, subjectively mild tissue reaction, may cause fairly strong acute inflammations when introduced into the loose connective tissue surrounding nerves [42]. The term ‘subjectively’ enters in the above sentence because biocompatibility is a relative concept which is determined by the risk–benefit ratio, which again may be different for different applications. Therefore, the use of the term bio-compatible should be used very cautiously since it may have misleading implications depending on the application. The reviews by Naahidi et al. [42] and Sundar et al. [43] should be referred to for more details on bio-compatible and biodegradable nanostructures.

Secondly, a drug carrier should be biodegradable. The carrier should be properly eliminated from the tissue after performing its function without requiring further treatment for removal. Even if it fulfills the requirements of a bio-compatible material, if it is not biodegradable, the drug carrier may trigger a reaction from the immune system or accumulate in organs such as the liver, kidney, and spleen, leading to toxic or potentially life-long side effects. Some examples of widely employed biodegradable polymers for drug delivery are PLGA, PLA (poly-lactic acid), and PCL (poly-ε-caprolactone) [44]. Well-known PEO-polypropylene oxide (PPO)-PEO tri-block copolymers made up of hydrophilic polyethylene oxide and hydrophobic polypropylene oxide groups (e.g., the Pluronic^®^ series) have also found wide use due to their excellent biodegradability, relatively small critical micelle concentrations, and better drug loading properties. Chitosan and Gelatin are examples of natural polymers which offer great biocompatibility and biodegradability.

**Figure 2:**
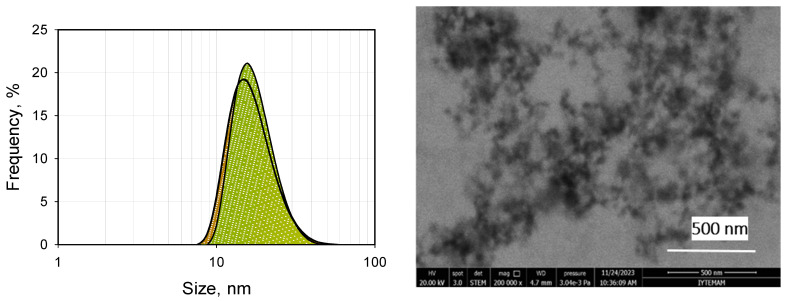
Size distribution by DLS (**left**) and STEM (**right**) images of 10^−3^ M P-123 (barren micelles).

**Figure 3:**
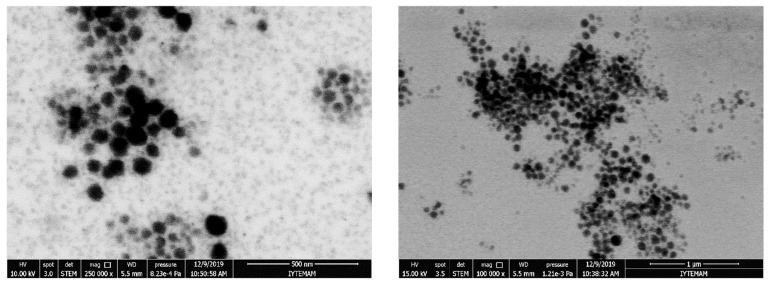
STEM images of curcumin-loaded P-123 micelles (**left**), probucol-loaded P-123 micelles (**right**).

Thirdly, the hydrophobic cores of the micelles must be large enough to be able to contain enough smaller drug molecules, while being small enough to penetrate the target cells. Micelles of copolymers and natural polymers with sizes in the order of 10–100 nm perfectly satisfy these criteria. Some examples of direct measurements carried out in our labs are given in Figure 2 and Figure 3. The left-hand-side graph in Figure 2 gives the in situ dynamic light scattering size distribution of barren micelles of the well-known Pluronic^®^ brand tri-block copolymer P-123 in distilled water. The figure shows that the micelles have an average size of around 18 and a maximum size of around 40 nm (DLS, dynamic light scattering). The average size observed agrees with the reported values in the literature [45,46]. A scanning transmission electron microscope (STEM) picture of the same micelles after immobilization on carbon grids is given in the right-hand-side graph of Figure 2. The image clearly shows the size and the spherical shape of the micelles. Scanning transmission electron microscope (STEM) images of the same micelles loaded with strongly hydrophobic drugs are presented in Figure 3.

Lastly and most importantly, the micelles must have sufficient stability to reach the target site intact and have sensitivity to respond to the target site for delivery of the load and display a controlled release profile. Placement of the active molecules within a micelle’s core is not sufficient for success in drug delivery for two main reasons. Micelle formation, which corresponds to a thermodynamic equilibrium at a specific surfactant concentration, is a reversible process with concentration changes. Considering that dilution of the micellar solution during intravenous injection is unavoidable, the stability of the drug-carrying micelles should be a serious concern in real applications. On the other hand, assuming that the micelles reach the target site intact, they must also show the proper response at the target site for the successful delivery of their chemical load. Therefore, the vast number of copolymeric building blocks available must be modified by some means to form micelles with proper stability and response characteristics to be able to function as dependable drug-delivery vehicles. Due to the large variety of options available, the routes for forming micelles like drug-delivery vehicles with different features can be classified under those which provide improved stability for successful transport and those which lead to the proper response at the target site.

In drug-delivery applications, the hydrophobic cores of the micelles of polymeric molecules act as a solvent for lipophilic drug molecules and store the drug until it is released at the target site [32,47,48,49,50,51,52,53,54,55,56,57,58,59]. Therefore, it is imperative that polymeric micelles should maintain their structural integrity and not disintegrate in an untimely manner in the bloodstream until they reach the target site. Unfortunately, deterioration during intravenous injection is a serious threat, since the micellar solutions necessarily become diluted below CMC in addition to being exposed to pH and salt changes and contact with native proteins and cells in body fluid [29,60,61].

To clarify the underlying causes of this instability, it is instructive to contrast the dominant stability mechanisms of natural and synthetic micelles. Hydrophobically modified chitosan micelles, as natural polymers in which N-acylation introduces long-chain hydrophobic substituents on amino groups, exhibit cooperative hydrophobic associations between these chains, which will be discussed in the following sections. This forms a cohesive, interlocked matrix that encapsulates lipophilic drugs and resists disassembly under dilution, since breakup would require simultaneous disruption of multiple hydrophobic contacts—an energetically unfavorable process. In contrast, synthetic block copolymer micelles such as Pluronic P-123 adopt a core–corona architecture, with a hydrophobic PPO core and a hydrophilic PEO corona. When the concentration falls below the CMC, favorable corona–water interactions drive de-association into unimers as the system returns to thermodynamic equilibrium, leading to dilution-induced disintegration.

Micelle stability can be examined from two perspectives: thermodynamic stability, which describes how the system acts as micelles are formed and reach equilibrium, and kinetic stability, which describes the behavior of the system over time in solution. The CMC is one of the parameters used to characterize the thermodynamic stability of micelles and is related to thermal energy and the effective interaction energy between polymers and bulk solution. Since polymer chains of polymeric micelles have more points of interaction, they have a lower CMC than the low-molar-mass surfactant micelles. It is believed that the lower the CMC value, the higher the micellization ability and micellar stability [3,33,62,63,64]. The length of the hydrophobic segment is also shown to be one of the factors affecting stability [65,66]. Other than these, various modifications can be devised to increase the thermodynamic stability of the micelles [64,67,68,69].

**Figure 4:**
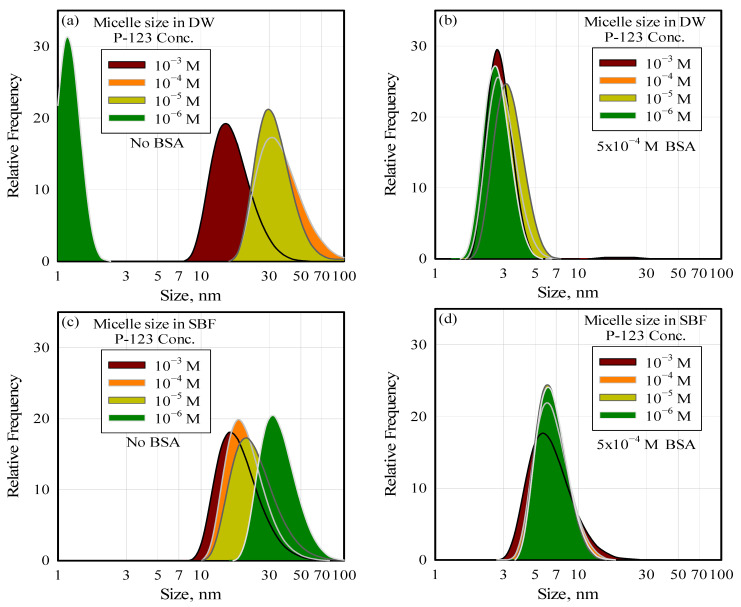
The effect of dilution and the presence of protein on the integrity (size) of the P-123 micelles in water (**a**,**b**) and in SBF (**c**,**d**).

The authors of this paper investigated the thermodynamic and kinetic stability of barren and drug-loaded Pluronic^®^ P-123 micelles in systematic re-dilution steps with DW and simulated body fluid (SBF). They observed through STEM, TEM, and DLS measurements that the drug-loaded micelles exhibited significant disintegration upon dilution in DW and SBF. The left-hand-side graphs in Figure 4 show the effect of dilution on barren micelles when they are diluted from a stock solution of full-grown micelles. The average micelle size at the solution surfactant concentration of 10^−3^ M (16 nm) became progressively smaller and reached a value close to that of the P-123 monomer (around 4 nm) at a surfactant concentration of 10^−7^ M P-123 following dilution. Disintegration due to dilution could not be prevented when the micelles were formed in SBF solutions or when they were loaded with lipophilic drug molecules. This behavior is consistent with the core–corona equilibrium mechanism outlined above, where corona–water interactions dominate near and below the CMC, favoring micelle disassembly.

Their more recent work conclusively demonstrates that native plasma components (in this case, the protein BSA) significantly affect micelle integrity. The graph on the left-hand side of Figure 4 shows how micelles are affected by BSA in solution. It can be observed that the average micelle size of 18 nm, in the absence of BSA, decreases with dilution, reaching less than 3 nm at a BSA concentration of 5 × 10^−4^ M. BSA-induced micelle disintegration also cannot be prevented in SBF or in the presence of encapsulated lipophilic drug molecules [47]. It should be noted that the BSA concentration at which the micelles disintegrate is within the range of the plasma protein concentration. It is suggested that dilution and the presence of native plasma components must be considered in devising micelles as drug carriers.

Micelle formation is a reversible process that is highly dependent on weak intermolecular interactions. These weak bonds can be strengthened by cross-linking strategies within the shell or core regions [10,70,71,72,73,74,75,76]. Wang et al. [77] state that redox-sensitive cross-linking of the micelles within the shell reduces their CMC and enhances their stability against severe conditions encountered during the intravenous injection. Similarly, Lee et al. [78] suggest pH-hydrolysable cross-linking within the shell improved the micellar physical stability even in the presence of micelle-disrupting surfactants, SDS. Lu et al. [71] list several covalent cross-linking methods in the shell or the core regions such as photo/ultraviolet-induced dimerization, di-functional cross-linkers, click cross-linking, silicon chemistry methods, and reversible boronate ester bonds. In addition, other cross-linking methods based on non-covalent molecular interactions, including static electric interaction and hydrogen bonding, have been applied to increase the stability of micelles [79]. However, shell cross-linking may alter the surface and hydrophilicity characteristics of the micelles, which in turn affect their solvation properties in the bloodstream.

The CMC is inversely proportional to the length of the hydrophobic block; as the chain becomes longer, the hydrophobic interactions within the micelle core strengthen, thereby lowering the CMC and enhancing micellar stability. To reduce CMC, hydrophobic interactions can be increased by adding hydrophobic segments to the micelle core. Similarly, the presence of highly hydrophobic drugs has been suggested to increase micelle stability due to hydrophobic interactions between the encapsulated drug and the polymer [29,80,81,82,83]. In a few cases reported in the literature, interactions between the encapsulated drug and the micellar core have been shown to lower the CMC and decrease the drug release rate. The authors also state that the methods applied to form micelles, such as dialysis and co-solvent evaporation, or changes in solvent conditions or temperature also have an effect on the stability of micelles. Another potential method for improving micelle stability of synthetic polymers is to wrap the drug-loaded micelles with a natural polymeric structure such as chitosan by cross-linking the chitosan molecules around the individual micelles [14].

Modifications for improving the response at the target site are carried out to provide the micelles with functionalities such as pH-, light-, or redox-responsive stimuli sensitivity, or charge-converted and core/shell cross-linked micelles for proper intracellular delivery at the target site [69,77,84,85,86,87,88,89]. Stimulus-sensitive polymeric micelles are customized to release drugs by changing their composition or conformation after exposure to extracellular or intracellular triggers. These structural changes can occur in different ways, such as by cleaving the specific linker between the transported substance and the polymer, cleaving the shielding polymer block, affecting changes in the charged groups on the polymer, or solubilizing the polymer [56,74,90,91]. There are excellent review papers [69,92] which summarize the details of stimuli-responsive polymeric micelles.

## 4. Natural Polymers (Biopolymers)

Micelles can also be prepared from natural biopolymers that are produced by the cells of living organisms and derived from a wide variety of sources, including plants, animals, and microorganisms. Due to their similarity to the extracellular matrix, mechanical tunability, high biocompatibility, and high water-holding capacity, biopolymers have been employed in a variety of biomedical applications such as pharmaceuticals, drug-delivery applications, tissue regeneration scaffolds, and imaging agents. They help prevent chronic inflammation, immune responses, and associated toxic effects [93,94,95], and their environmental impact is minimal (during production and application) [96,97].

Natural biopolymers can be generally grouped into three origins: polysaccharides, proteins, and microbially fermented biopolymers [96] (Table 1). They may originate from plants, animals (terrestrial or marine), or algae, or be produced through microbial fermentation. Cellulose, starch, pectin, and mannan are polysaccharides originating from plants, whereas chitin, heparin, and hyaluronan are animal-based polysaccharides. Agarose, alginate, carrageenan, and fucoidan are non-animal groups of polysaccharides extracted from algae. A brief description of each group along with its best-known, most used principal member will be presented in the following paragraphs for completeness. For a more detailed discussion, refer to the recent papers by O’Brien [94], Garg et al. [98], Nikolova and Chavali [99], Qu et al. [100], Shick et al. [101], and Williams [102], which provide extensive reviews on natural materials.

**Table 1:** Classification of some common natural biomaterials.

Type	Origin	Examples	References
Polysaccharides	Plant	Cellulose, Starch, Pectin, Mannan	[103,104,105,106,107,108,109,110]
	Animal	Chitin/Chitosan, Heparin, Hyaluronan	[20,64,85,111,112,113,114]
	Microbial	Dextran, Pullulan	[84,115,116,117,118,119,120]
	Algal	Fucoidan, Agar/Agarose, Carrageenan, Alginate	[121,122,123,124,125,126,127,128]
Proteins	Animal	Collagen, Gelatin, Fibrin, Silk	[129,130,131,132,133,134,135,136,137]
Microbially fermented	Microbial	Polyhydroxy-alkanoates	[138,139,140,141]

## 5. Modification of Biopolymers for Micellization to Use as Drug Delivery Vehicles

Manufacturing biopolymeric structures homogeneously and reproducibly, especially in specific forms such as spherical nanoparticles, films, foams, or scaffolds, is a hot field of recent research. The micelles of biopolymers have also started to attract significant interest in recent years as drug-delivery agents in the pharmaceutical field. However, most of the time a biopolymer must be subjected to some form of modification to form micelles with specific properties in order to be able to find a wide application area in drug delivery and tissue engineering [142,143,144,145].

**Figure 5:**
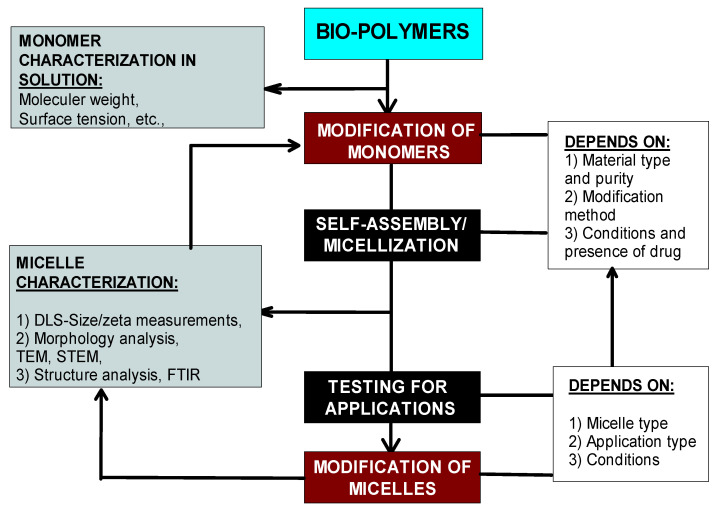
The general methodology of biopolymer modification and its relation to micellization.

Specifically, biopolymers in their natural form are not suitable for self-assembly; micelle formation requires assistance through the chemical modification of biopolymer molecules to obtain hydrophobic functional groups. Once they are made to self-assemble, biopolymeric micelles present great potential for controlled release, drug targeting, and hydrophobic active material solubilization with the added advantage of intrinsic biocompatibility and biodegradability, which is always a question with synthetic polymers. The important steps/factors involved in their modification and micellization is generalized by the following flowsheet (Figure 5).

Biopolymers can be modified through various methods depending on their functional groups. Uronic acid-rich polysaccharides like alginate are typically modified via carboxyl group amidation or esterification, while aminated polysaccharides undergo modifications such as quaternization, N-acylation, or N-alkylation [146,147,148,149,150]. The following summarizes key studies on the modification and micellization of widely used biopolymers.

Cellulose possesses rich functional groups, allowing for self-assembly through modifications including esterification [151,152,153,154,155,156,157,158], etherification [103,159], backbone grafting [160,161,162,163], ROP [162,164,165], ATRP [166,167,168], and RAFT polymerization [169,170,171]. Stimuli-responsive self-assembly has been achieved by tuning molecular structures or external conditions like temperature [172,173,174], light [74,175], pH [176], or multiple triggers [177,178]. For example, pH-responsive micelles have been formed from HEC-graft poly(acrylic acid) [179], and pH-sensitive molecular brushes have also been reported.

Amphiphilic cellulose derivatives have been developed to self-assemble into micelles or vesicles [180,181], with sizes ranging from 20 to 430 nm [103]. These include QC-g-OCL polymers, cellulose-g-PCL systems, and cellulose derivatives carrying long alkyl chains for hydrophobic drug encapsulation [57,104,105].

Heparin-based micelles have been used for the delivery of growth factors and anticancer drugs [182,183,184]. Systems include Tetronic-PCL-heparin micelles (CMC: 0.11 g/L, size ~114 nm), docetaxel-loaded pH-sensitive micelles with deoxycholate, and redox-sensitive micelles synthesized using heparin, β-sitosterol, and cysteamine (encapsulation efficiency: 58.47%) [85]. Other examples involve GA-loaded micelles [114] and heparosan-cholesterol nanocarriers for improved drug uptake [113].

Dextran derivatives have been modified with aldehydes, thiols, acrylates, and other functional groups for drug delivery [116,120,185]. Dextran-based amphiphilic copolymers using PLGA, PCL, and lipids have been developed for loading doxorubicin, docetaxel, and curcumin [186,187,188,189,190,191]. Functionalization with folic acid, cholesterol, and N-isopropylacrylamide has enhanced targeting [84,117,185,192,193]. Synthesis strategies include thiol–disulfide exchange [186], click chemistry [187], and end-to-end coupling [188]. Zwitterionic dextran micelles have also been reported [189].

Alginate, a hydrophilic polymer, requires hydrophobic modification for micelle formation. This has been achieved using long alkyl chains [194,195,196], esterification [197,198], and graft polymerization. Hydrophobic modified alginate was also synthesized by derivatization of sodium alginate with dodecyl glycidyl ether in an aqueous solution [86,199,200] and by esterification of octadecyl chains onto the polysaccharide backbone [201] in a different study. Modified alginate forms micelles with CMCs as low as 0.024 g/L [202] and shows aggregation at concentrations as low as 0.1 mg/mL [203]. Examples include alginate–curcumin micelles for hepatocyte targeting (size ~235 nm, zeta potential −29 mV) [119], calcium-crosslinked spherical micelles (CMC: 0.2 mg/mL) [128], and amphiphilic graft copolymers formed via living radical polymerization [202].

Chitosan’s abundant –NH_2_ and –OH groups allow for easy chemical modification, affecting its physicochemical and biological properties, particularly its solubility [204,205]. Modifications usually occur at the 2-NH_2_ position (N-substitution) due to its higher reactivity over the 3-OH and 6-OH groups [206]. Acylation reactions target NH_2_ (N-acylation), OH (O-acylation), or both (N,O-acylation), using reagents like acyl halides and acid anhydrides in solvents such as pyridine and methanol/water/acetic acid [146,147,149,150,207]. O-acylation requires NH_2_ protection, often attained using methanesulfonic acid [208], followed by deprotection for N,O-acylation [209].

Alkylation involves Schiff base reactions with aldehydes or ketones [210,211,212], forming –C=N bonds linked to bioactivities [213]. N-alkylation occurs on C2–NH_2_, and O-alkylation on C3 or C6–OH groups. Due to nucleophilicity, N-alkylation is generally favored. Reaction pathway selection depends on desired functionality, as –NH_2_ modification influences bioactivity, including antimicrobial properties [214]. Protection/deprotection strategies help preserve chitosan’s biodegradability and safety [205].

Chitosan-based micelles have been developed for hydrophobic drug delivery. Emami et al. [67] synthesized tocopheryl succinate-grafted chitosan oligosaccharide loaded with paclitaxel, enhancing micelle stability and reducing size. Huo et al. [215] created N-octyl-O-glycol chitosan (OGC) micelles for paclitaxel delivery with CMC values between 5.3 and 32.5 mg/L. Guo et al. [216] developed Gal-OCMC-g-SA micelles (160 nm, CAC 0.047 mg/mL) for liver-targeted docetaxel release. Another study used chitosan grafted with deoxycholic acid and N-acetyl-L-cysteine to load quercetin [209]. Jiang et al. [217] combined COS-DOCA and mPEG-PDLLA to produce paclitaxel-loaded mixed micelles (40 nm, 97.09% encapsulation). Examples of chitosan-based micelles developed as drug-delivery systems are summarized in Table 2.

Oral delivery applications also utilize chitosan’s mucoadhesive properties, interacting with mucin via electrostatic and hydrophobic effects [218,219]. Examples include paclitaxel [220], linoleic acid-grafted chitosan oligosaccharide micelles for docetaxel [221], and pH-sensitive N-naphthyl-N,O-succinyl chitosan micelles for meloxicam [222].

Other polysaccharides have also been explored. Pullulan, a microbial exopolysaccharide, has been used in various micelle systems: cholesterol-bearing pullulan [223,224,225,226,227,228], acetylated pullulan [220], poly(L-lactide)-grafted pullulan [118,221], pullulan–desoxycholic acid–PEI [222], and PLGA–grafted pullulan [223]. Galactosylated pullulan-curcumin conjugates have shown targeted delivery to hepatocarcinoma cells [119], and pullulan–tocopherol succinate–folic acid micelles (149.5 nm, –49 mV, CMC: 194.87 μg/mL) were used for epirubicin delivery to Hela and MCF-7 cells [224]. Reduction-sensitive pullulan–stearic acid micelles have also been used for intracellular doxorubicin [225,226,227,228].

**Table 2:** Drug-loaded chitosan-based micelles and their important properties.

Name of Amphiphilic Copolymer Micelle	Drug/ Molecule	Size (nm)	ZP (mV)	EE (%)	CMC (mg/mL)	Ref.
*Stearyl-grafted* chitosan	Atorvastatin	97.19	−8.27	10.4–35.0	6.9 × 10^−3^–21 × 10^−3^	[64]
N-phthaloyl-carboxymethyl chitosan	L floxin and BSA	60–90 30–200	-	8.5 52.0	0.20	[112]
*Cholesteryl hemisuccinate (CHS)*-conjugated chitosan	Docetaxel	303	+21.3	NA	NA	[229]
*Stearic acid-grafted* chitosan oligosaccharide	Docetaxel	20.4	+53.1	55.0	0.022	[68]
Water-soluble *N-palmitoyl* chitosan	Ibuprofen	~150.0	-	~50.0	2.0 × 10^−3^–37.2 × 10^−3^	[212]
Folate-modified *N*-Succinyl-*N*′-Octyl Chitosan	10 Hydroxyca-camptothecin	100–200	−20.0 to +38.0	57.0–58.0	-	[230]
N-succinyl-N′-octyl chitosan micelles	Docetaxel	100–200	-	36.4 ^a^	5.9 × 10^−3^– 3.1 × 10^−2^	[231]
Fatty acid grafted chitosan-based copolymer micelles	Cefiximetrihy-drate	520	+42	38–52	NA	[111]
*Grafting oleic acid (OA)* on the chitosan (CS) skeleton and *penetrating (PEN)* and *(MAN)* conjugation.	pVGF	199.8 ± 15.7 nm	Positive	NA	NA	[232]
Redox-sensitive chitosan derivative (y *cholesterol, sulfhydryl*, and *mPEG (mPEG-CS(SH)-CHO))*	Paclitaxel	158	+26.9	88.3	NA	[233]
*O-methyl-O′-succinylpolyethylene glycol*- and *oleic acid*-grafted chitosan	Camptothecin	140 nm	Positive	78	0.150–0.147 0.076–0.065	[234]
LA–CMCS (Linoleic acid–carboxymethyl chitosan)	Paclitaxel	93–119	−16 to −29	56–67	~11–18 × 10^−3^	[235]
CS–g–OA (Oleic acid-grafted chitosan)	Coumarin-6	335.5/491	+20.5 to +38.5	29	0.5748	[236]
CS–SA–DA (Succinic anhydride and deoxycholic acid-modified chitosan)	Curcumin	228/269	−44 to −29	80.8	0.093	[237]
LCNE–LA (Low-MW chitosan–nicotinic acid–lipoic acid conjugate)	Doxorubicin	218/254	+26/35.2	92	0.1808	[238]

EE: encapsulation efficiency; ZP: zeta potential; BSA: bovine serum albumin; ^a^: Loading capacity.

Hydroxyethyl starch (HES), a semi-synthetic polysaccharide, has been esterified with fatty acids (lauric, palmitic, stearic) to form micelles and vesicles (20–350 nm) [106]. Thermo-responsive starches (HBPS), synthesized with butyl glycidyl ether, exhibit temperature-dependent drug release [107]. Amylopectin modified with poly(lactic acid) also forms micelles (20.7–77.2 nm) with adjustable CAC values (0.038–0.190 mg/L) [108].

Importantly, modifying natural polymers like chitosan can significantly alter their properties. For instance, modifying –NH_2_ groups may reduce pH sensitivity and positive surface charge, affecting bioactivity and drug-delivery performance [239].

## 6. Characterization of Biopolymers and Their Micelles

Biopolymers, whether in their native (unmodified) or chemically modified forms—as well as the micelles formed from them—are characterized using a range of analytical techniques. These characterizations are essential not only to confirm molecular structure but also to assess surface activity, which plays a crucial role in micelle formation and functionality.

Structural characterization of both unmodified and modified biopolymeric molecules is typically conducted using techniques such as elemental analysis, potentiometric titration, ninhydrin assay, ^1^H and ^13^C NMR, HSQC-NMR, Fourier-transform infrared spectroscopy (FTIR), matrix-assisted laser desorption/ionization mass spectrometry (MALDI-MS), and thermogravimetric analysis (TGA). For modified biopolymers intended for micelle formation, surface activity characterization is also necessary. Common methods employed for this purpose include surface and interfacial tension measurements, contact angle analysis, and surface energy determination [239,240,241].

Once micelles are formed, further characterization is required to determine their morphological and surface properties. In addition to the aforementioned techniques, specialized tools such as small-angle X-ray scattering (SAXS), dynamic light scattering (DLS), atomic force microscopy (AFM), scanning electron microscopy (SEM), transmission electron microscopy (TEM), and scanning transmission electron microscopy (STEM) are routinely used for comprehensive micellar characterization [239].

**Figure 6:**
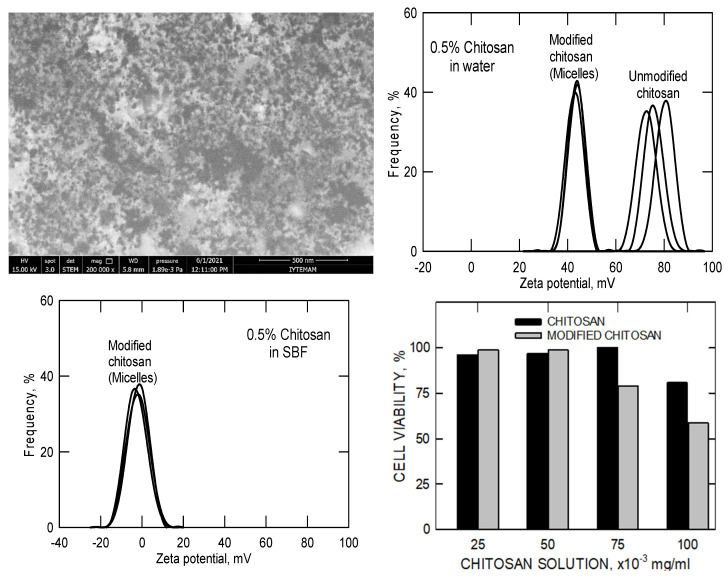
STEM pictures, zeta potentials, and biocompatibility results of the unmodified (pristine) and modified (hydrophobisized) chitosan molecules in water and in simulated body fluid. (The multiple black lines represent 3 repeats of the same measurements).

In Figure 6, STEM images clearly reveal the formation of spherical, non-porous chitosan micelles with diameters between 30 and 50 nm in aqueous media. Given the relatively high molecular weight of the modified chitosan (~500,000 g mol^−1^), these structures likely represent coiled aggregates of hydrophobically modified chitosan chains, possibly intercalated with drug molecules. This self-assembly behavior contrasts with more conventional polymeric micelles such as those formed from Pluronic P-123, which typically exhibit smaller diameters (~20 nm) (Figure 2 and Figure 3).

The corresponding zeta-potential and cell-viability data further clarify the physicochemical and biological implications of this hydrophobic modification. In aqueous solution, pristine chitosan displays a high positive zeta potential (≈70–90 mV) due to the abundance of protonated amino groups. After partial acylation, the modified chitosan micelles maintain a positive surface charge (≈40–50 mV), indicating that a substantial portion of amine groups remains unreacted and available for electrostatic interactions. When dispersed in simulated body fluid (SBF), the zeta potential shifts toward neutrality and the distribution broadens, reflecting ionic adsorption and screening of surface charges by physiological electrolytes. Although this reduction diminishes colloidal repulsion, it reflects realistic physiological conditions and is characteristic of chitosan-based micellar systems. The accompanying biocompatibility results show that both pristine and modified chitosan maintain high cell viability (>80%) at concentrations up to 50 × 10^−3^ mg mL^−1^, with only a slight decrease for the hydrophobically modified samples at higher concentrations. Together, these results demonstrate that partial hydrophobic substitution promotes micellization without compromising biocompatibility, while the remaining positive surface charge contributes to colloidal stability and facilitates potential electrostatic drug loading near physiological pH.

The Krafft temperature is defined as the minimum temperature at which the solubility of an ionic surfactant becomes equal to its critical micelle concentration (CMC); below this temperature, micelles cannot form because the surfactant exists predominantly in a crystalline or precipitated state [242]. This concept originated from studies on low-molecular-weight ionic surfactants that exhibit sharp phase transitions between crystalline and micellar states. In contrast, polymeric or nonionic amphiphiles, such as hydrophobically modified chitosan, do not display a distinct Krafft point. Instead, micellization in these systems is governed mainly by polymer chain mobility, segmental flexibility, and solvent interactions rather than by solubility limits. In the same study [239,241], the micellar dispersions exhibited a pH of approximately 7.0 and a conductivity of 1.51 mS cm^−1^, consistent with near-neutral, moderately ionic conditions. Although the literature contains limited information on the Krafft temperature of chitosan-based micelles, available data suggest that their effective micellization temperature in simulated body fluid lies below 37 °C. This implies that chitosan micelles remain stable under physiological conditions, retaining their self-assembled structure during circulation and interaction with biological fluids.

## 7. Stability of Natural Polymeric Micelles

**Figure 7:**
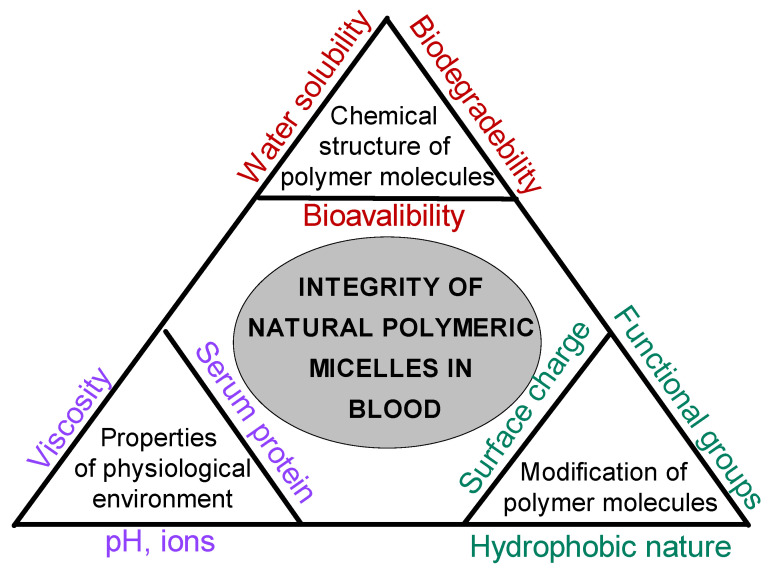
Factors influencing the integrity of biopolymeric micelles in physiological environments.

The possible factors, such as chemical structures of molecules, properties of the physiological environment, and the modification type of molecules, that affect the integrity of natural polymeric micelles in blood are summarized in Figure 7. In particular, hydrophobically modified chitosan systems benefit from cooperative hydrophobic association within the shell/core network, whereas classical block-copolymer micelles remain governed by core–corona equilibrium near the CMC.

The stability of biopolymeric micelles depends on multiple interrelated factors. Therefore, the use of bio-micellar systems in biological applications such as drug delivery requires additional care to take all the changes in the structure into account. One cannot use a single molecule as a reference anymore and expect similar properties from the micelles formed. The properties of micelles could be quite different depending on all the factors mentioned above.

Though a few studies have focused on the stability of the biopolymeric micelles and reported positive findings, the stability of the loaded biopolymeric micelles is usually an issue in the laboratory evaluation of the efficacy of delivery vehicles. Emami et al. [67] have shown that the physical incorporation of α-tocopherol succinate in chitosan-derived polymeric micelles for paclitaxel delivery enhances micelle stability. The improvement is due to the increased hydrophobic interaction between paclitaxel and the micellar core. In their study, to test their stability, micelles were stored at 4 °C for at least three months and their sizes were measured. The authors showed that the incorporation of α-tocopherol succinate into the micellar system produced smaller particles with high stability during storage without affecting the entrapment efficiency.

In another study, Mekhail et al. [64] synthesized and characterized stearyl chitosan and sulfated stearyl chitosan amphiphilic block copolymers. They have shown that polymeric micelles with or without drugs have a negative charge due to the negatively charged stearyl and sulfate groups that prevent the positive charge of the amino groups of chitosan. They concluded that the negative charge of polymeric micelles contributes to the stability of the colloidal micelle solution as a result of electrostatic repulsive forces between the micelles. Ye et al. [68] modified the core of amphiphilic *stearic acid-grafted* chitosan oligosaccharide (CSO-SA) micelles by the physical solubilization of stearic acid to reduce the burst drug release and enhance the physical stability of CSO-SA micelles. Zhu et al. [230] produced folate-modified N-succinyl-N′-octyl chitosan micelles (folate-SOC) for targeted delivery of 10-Hydroxycamptothecin. The stability of these micelles was tested by suspending these structures in PBS for 30 days and was then characterized. These results showed that folate-modified micelles display better storage stability. Zhu et al. [243] produced water-insoluble anticancer drug gambogic acid-loaded chitosan-based micelles. Their storage stability was tested using lyophilized samples kept at 4 °C for 2 months and they were found to be stable for up to 2 months.

Despite such findings which report positive stability data on biopolymeric micelles, it should be said that the deleterious effects of dilutional factors and the encounter with native blood components that the micelles must endure during intravenous injection must still be considered. The implications of this issue, which were reported by Polat and Polat [14,15,47], have been discussed in detail in their recent ongoing studies [239,240,241].

**Figure 8:**
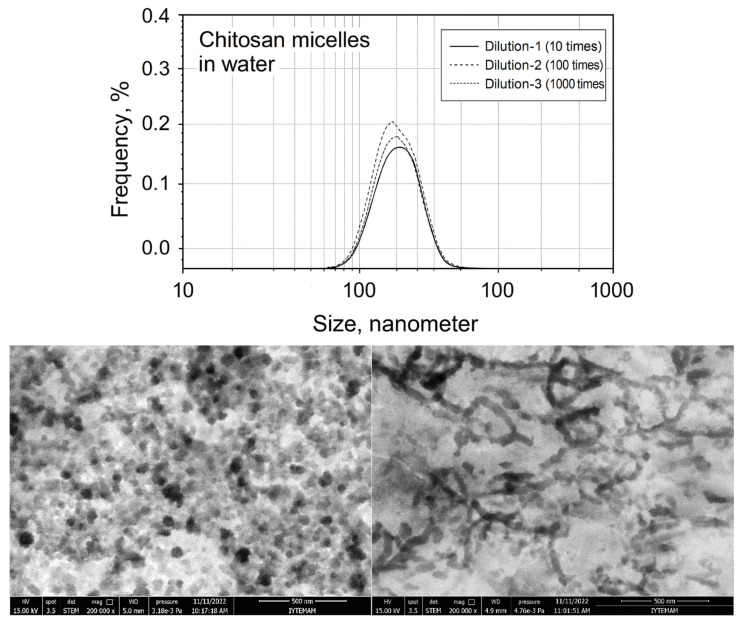
Dilution-induced disintegration of lipophilic drug-loaded chitosan micelles (**upper**) and STEM images of drug-loaded chitosan micelles in SBF before dilution (**bottom**, **left**) and after 1000 rounds of dilution (**bottom**, **right**).

The authors of this review paper studied the stability of chitosan micelles with dilution and protein interaction by DLS (dynamic light scattering) and the results are presented in Figure 8. It can be seen that the sizes of chitosan micelles do not change even after 1000 rounds of dilution in comparison to P-123 micelles in the case of dilution. However, the structure of natural polymeric micelles becomes unstable in body fluids upon protein interactions.

**Figure 9:**
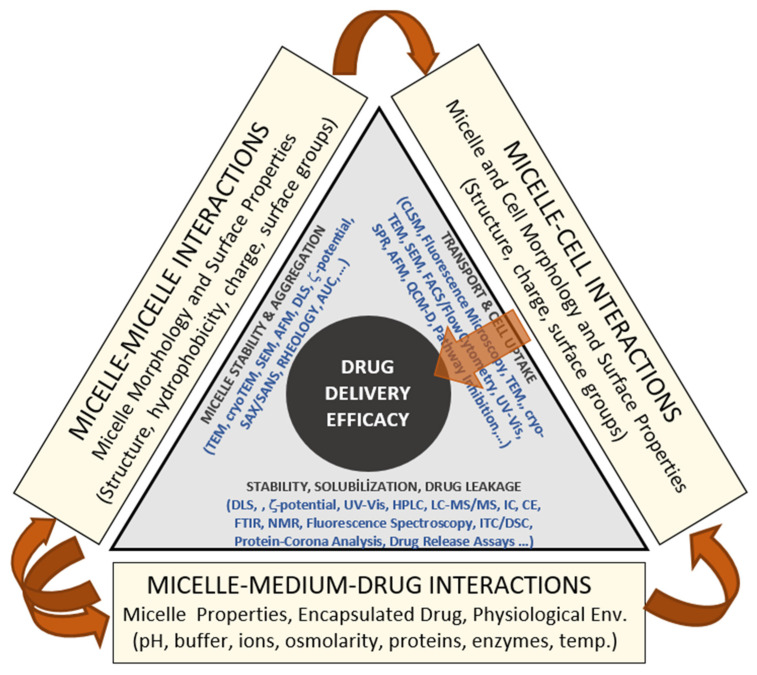
Conceptual framework linking micellar physicochemical behavior, biological interactions, and environmental stability to overall drug-delivery efficacy.

Figure 9 presents a conceptual model summarizing the complex interplay among micelle–micelle, micelle–cell, and micelle–medium–drug interactions that collectively determine drug-delivery performance under physiological conditions. The diagram is not intended as a quantitative or computational model but as a visual framework integrating the key physicochemical and biological factors that control the stability and efficacy of polymeric micellar systems. Along each axis, the figure identifies the dominant interaction modes governing system behavior. The base of the triangle represents micelle–medium–drug interactions, emphasizing the combined influence of environmental conditions (pH, ionic strength, osmolarity, proteins, enzymes, and temperature) and drug–micelle partitioning on colloidal stability, solubilization, and release kinetics. The left axis corresponds to micelle–micelle interactions, where variations in structure, surface charge, and hydrophobicity determine the extent of aggregation or dispersion phenomena typically characterized by techniques such as DLS, ζ-potential, SAXS/SANS, and microscopy. The right axis represents micelle–cell interactions, highlighting how micellar surface properties control cellular attachment, uptake, and biocompatibility, which are experimentally assessed by CLSM, FACS, or cell-viability assays. At the center lies drug-delivery efficacy, reflecting the integrated outcome of these coupled interactions. The model illustrates that premature micelle disintegration, excessive aggregation, or insufficient cell interaction can each compromise delivery performance. Conversely, achieving a balanced interplay among these three domains yields stable, biocompatible micellar systems capable of sustained and targeted drug release. Overall, Figure 9 provides a unified conceptual perspective linking physicochemical stability, environmental responsiveness, and biological interaction to therapeutic performance, thereby guiding the rational design of micellar nanocarriers for clinical use.

## 8. Conclusions

This review has examined the self-assembly behavior of both synthetic and natural polymers into micellar structures capable of encapsulating hydrophobic (water-insoluble) drugs, with a particular focus on their applicability in pharmaceutical drug-delivery systems. Polymeric micelles offer significant advantages for drug delivery, including enhanced cellular uptake efficiency resulting from their nanoscale dimensions. They also exhibit favorable biocompatibility and biodegradability, making them attractive candidates for pharmaceutical applications.

However, natural polymers typically require chemical modification to facilitate self-assembly and micelle formation. The efficiency of micellization and the resulting physicochemical properties of the micelles are strongly influenced by the type and degree of chemical modification applied. Importantly, these modifications may alter key intrinsic properties of the natural polymers—such as pH responsiveness, exemplified by chitosan—which must be carefully considered in the design of drug-delivery systems. Based on the literature reviewed, several key conclusions can be drawn regarding micellar systems that encapsulate drugs through physical entrapment (i.e., without covalent bonding):

- Synthetic polymeric micelles generally lack stability under physiological dilution conditions, leading to disassembly upon administration.
- Natural polymeric micelles tend to exhibit improved stability under dilution compared to synthetic counterparts; however, they remain susceptible to destabilization upon interaction with serum proteins.
- Both synthetic and natural polymeric micelles experience structural compromise in the presence of blood proteins, which poses a significant challenge for systemic administration.

In light of these findings, it is recommended that future research focus on strategies to enhance micelle stability in biological environments. Approaches such as surface modification, cross-linking, or the incorporation of protective coatings may enhance micellar integrity during circulation and prior to cellular uptake.

From a broader conceptual standpoint, the current state of micellar research reveals a transition from empirical formulation toward mechanism-driven design. Future progress will depend on integrating kinetic modeling, interfacial thermodynamics, and in situ characterization techniques to predict and control micelle behavior under physiological conditions. Developing quantitative frameworks that link micelle composition, stability, and drug-release kinetics will be essential for rational optimization. Moreover, the convergence of polymer chemistry, computational modeling, and biophysics is expected to accelerate the development of micelle systems tailored for specific therapeutic routes, oral, transdermal, and intravenous, bridging fundamental science with clinical utility.

These findings highlight that while achieving structural stability under physiological conditions remains a major design challenge, translating these advances into practical therapeutic systems is the next logical step toward real-world application. It should also be noted that comparisons between synthetic and natural polymeric micelles are not always straightforward. The stability and assembly behavior of each system depend strongly on the polymer’s intrinsic molecular parameters, such as molar mass, block-length ratio, degree of hydrophobic modification, and the chemistry of pendant groups, rather than solely on its synthetic or biological origin. Therefore, when assessing or comparing micellar stability, these specific structural features must be considered alongside polymer type, as they often play the dominant role in determining micelle architecture and robustness under physiological conditions.

In recent years, several polymeric micelle systems have progressed from laboratory studies to clinical evaluation. Formulations based on PEG-PLA and PEG-PCL have entered clinical trials for anticancer drugs such as paclitaxel, doxorubicin, and cisplatin, where they have shown improved solubility, reduced toxicity, and better pharmacokinetic profiles. Natural polymer-based micelles, particularly those derived from chitosan, alginate, and fucoidan, are also drawing attention for their biocompatibility and additional biological functions such as mucoadhesion and site-specific targeting. These features make them promising candidates for further clinical translation. Looking ahead, it is expected that hybrid micellar systems combining the structural control of synthetic polymers with the bioactivity of natural materials will play a key role in developing safer and more effective nanomedicines.
